# SoxD transcription factor deficiency in Schwann cells delays myelination in the developing peripheral nervous system

**DOI:** 10.1038/s41598-021-93437-9

**Published:** 2021-07-07

**Authors:** Ella Ittner, Anna C. Hartwig, Olga Elsesser, Hannah M. Wüst, Franziska Fröb, Miriam Wedel, Margit Schimmel, Ernst R. Tamm, Michael Wegner, Elisabeth Sock

**Affiliations:** 1 Institut für Biochemie, Emil-Fischer-Zentrum, Friedrich-Alexander-Universität Erlangen-Nürnberg, Fahrstrasse 17, 91054 Erlangen, Germany; 2 Institut für Humananatomie und Embryologie, Universität Regensburg, Regensburg, Germany

**Keywords:** Neuroscience, Development of the nervous system, Glial biology, Gliogenesis, Myelin biology and repair, Peripheral nervous system, Developmental biology, Differentiation

## Abstract

The three SoxD proteins, Sox5, Sox6 and Sox13, represent closely related transcription factors with important roles during development. In the developing nervous system, SoxD proteins have so far been primarily studied in oligodendroglial cells and in interneurons of brain and spinal cord. In oligodendroglial cells, Sox5 and Sox6 jointly maintain the precursor state, interfere with terminal differentiation, and thereby ensure the proper timing of myelination in the central nervous system. Here we studied the role of SoxD proteins in Schwann cells, the functional counterpart of oligodendrocytes in the peripheral nervous system. We show that Schwann cells express Sox5 and Sox13 but not Sox6. Expression was transient and ceased with the onset of terminal differentiation. In mice with early Schwann cell-specific deletion of both Sox5 and Sox13, embryonic Schwann cell development was not substantially affected and progressed normally into the promyelinating stage. However, there was a mild and transient delay in the myelination of the peripheral nervous system of these mice. We therefore conclude that SoxD proteins—in stark contrast to their action in oligodendrocytes—promote differentiation and myelination in Schwann cells.

## Introduction

Rapid saltatory conduction is key to efficient information processing in the vertebrate nervous system and requires segmental ensheathment of axons by myelin. Myelin sheaths are formed as specialized organelles by oligodendrocytes in the central nervous system (CNS) and Schwann cells in the peripheral nervous system (PNS). The regulatory networks that drive development and myelination in both cell types have been intensely studied over the last decade^1,2^. Several transcription factors have been identified as key components with diverse functions ranging from determination of cellular identity, preservation of the precursor state, induction of proliferation or cell cycle exit to prevention or promotion of terminal differentiation.

Sox5, Sox6 and Sox13 make up the SoxD subgroup of Sox transcription factors^3^. Like all Sox transcription factors, SoxD proteins possess a high-mobility-group (HMG) domain as their DNA-binding domain. Additionally, they share other subgroup-specific functional domains such as a leucine zipper for dimer formation^4^. All three SoxD proteins are present in oligodendroglial cells already at the time of specification. Expression of Sox5 and Sox6 lasts until terminal differentiation, whereas Sox13 expression persists into the differentiated state^5,6^. In oligodendroglial cells, SoxD proteins function in a largely redundant manner, with Sox6 contributing most and Sox13 contributing least to SoxD activity^5–7^. Mice deficient for Sox5 and Sox6 exhibit altered migration and a precocious differentiation of oligodendroglial precursors arguing that SoxD proteins keep oligodendroglial cells in the precursor state, promote their migratory activity and inhibit terminal differentiation^5,7^.

So far, no data exist on the role of SoxD proteins in Schwann cells and in PNS myelination. Schwann cells are derivatives of the neural crest and ontogenetically closely related to melanocytes. Considering that Sox5 has been detected in pre-migratory and migratory cells of the cranial neural crest, in glial cells of cranial ganglia and in melanocytic cells^8–10^, it seemed reasonable to assume that SoxD proteins are also expressed and functional in Schwann cells. To test this hypothesis, we first determined the expression pattern of SoxD proteins in the Schwann cell lineage and then analysed mouse mutants with SoxD deficiencies to study their role in Schwann cell development and myelination.

## Results

### Sox5 and Sox13 proteins are transiently expressed in developing Schwann cells

**Figure 1:**
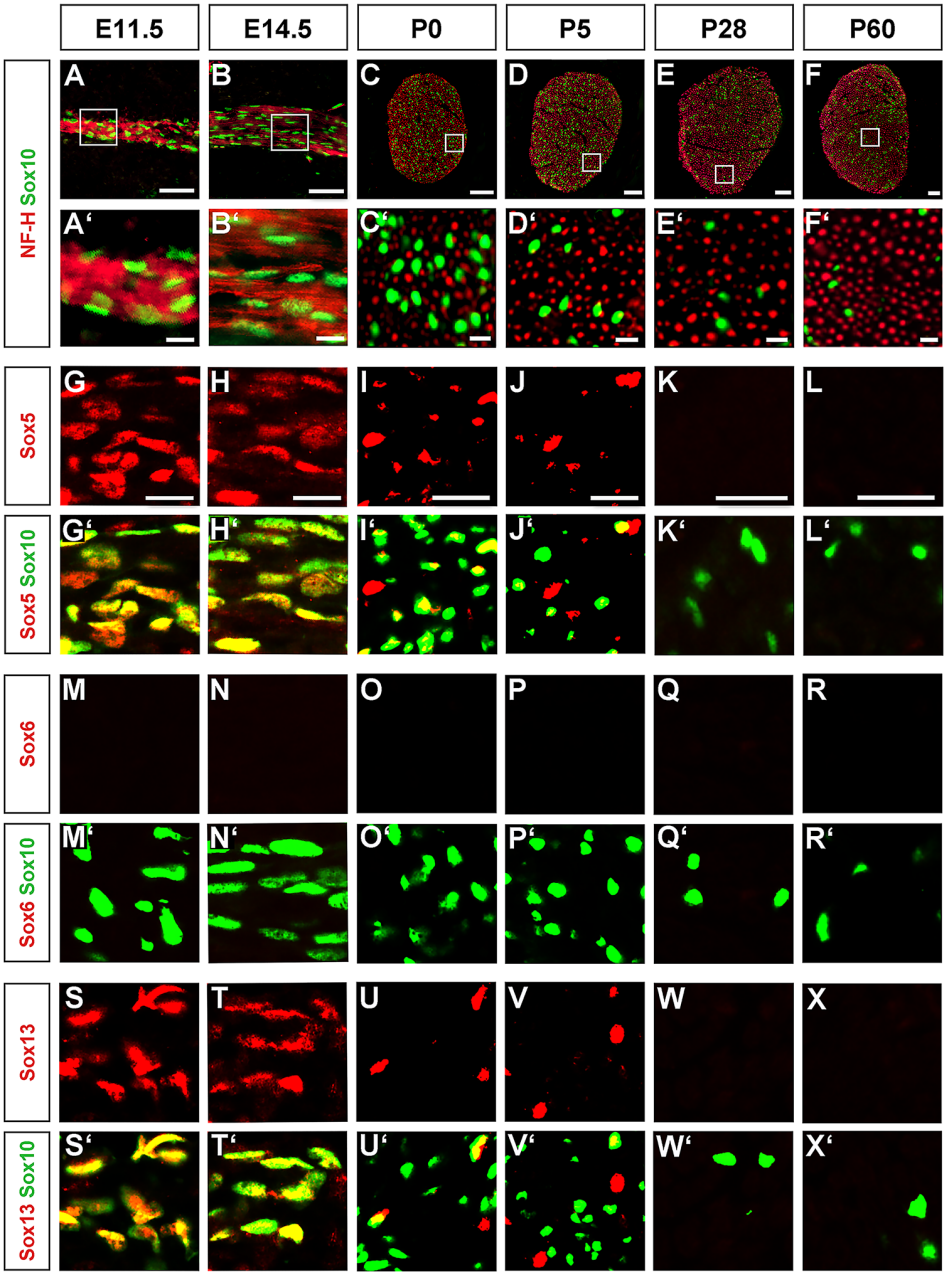
Analysis of SoxD expression in developing and adult Schwann cells. After localization of peripheral nerves by neurofilament staining (**A–F,A′–F′**, in red), co-immunohistochemistry was used to determine expression of Sox5 (**G–L**), Sox6 (**M–R**) and Sox13 (**S–X**) (all in red) in Sox10-positive Schwann cells (**A–F,A′–F′,G′–L′,M′–R′,S′–X**′, in green) of spinal nerves (**A,A′,B,B′,G,G′,H,H′,M,M′,N,N′,S,S′,T,T′**) and sciatic nerves (**C–F,C′–F′,I–L,I′–L′,O–R,O′–R′,U-X,U′–X′**) at E11.5 (**A,A′,G,G′,M,M′,S,S′**), E14.5 (**B,B′,H,H′,N,N′,T,T′**), P0 (**C,C′I,I′,O,O′,U,U′**), P5 (**D,D′,J,J′,P,P′,V,V′**), P28 (**E,E′,K,K′,Q,Q′,W,W′**) and P60 (**F,F′,L,L′,R,R′,X,X′**). Boxes in (**A–F**) mark the areas from which magnifications were taken for the other panels on parallel sections. Scale bars, 50 µm in (**A–F**), 10 µm in (**A′–F′**), 25 µm in (**G–L**).

To analyse SoxD expression in developing and mature Schwann cells, we performed immunohistochemical stainings on spinal nerves at E11.5 and E14.5 as well as on sciatic nerves at P0, P5, P28 and P60 with antibodies directed against Sox5, Sox6 and Sox13. Spinal and sciatic nerves were visualized by neurofilament staining (Fig. 1A–F,A′–F′). Within the nerves, nuclei were stained with antibodies against Sox10 to identify Schwann cells (Fig. 1A–F, A′–F′).

**Figure 2:**
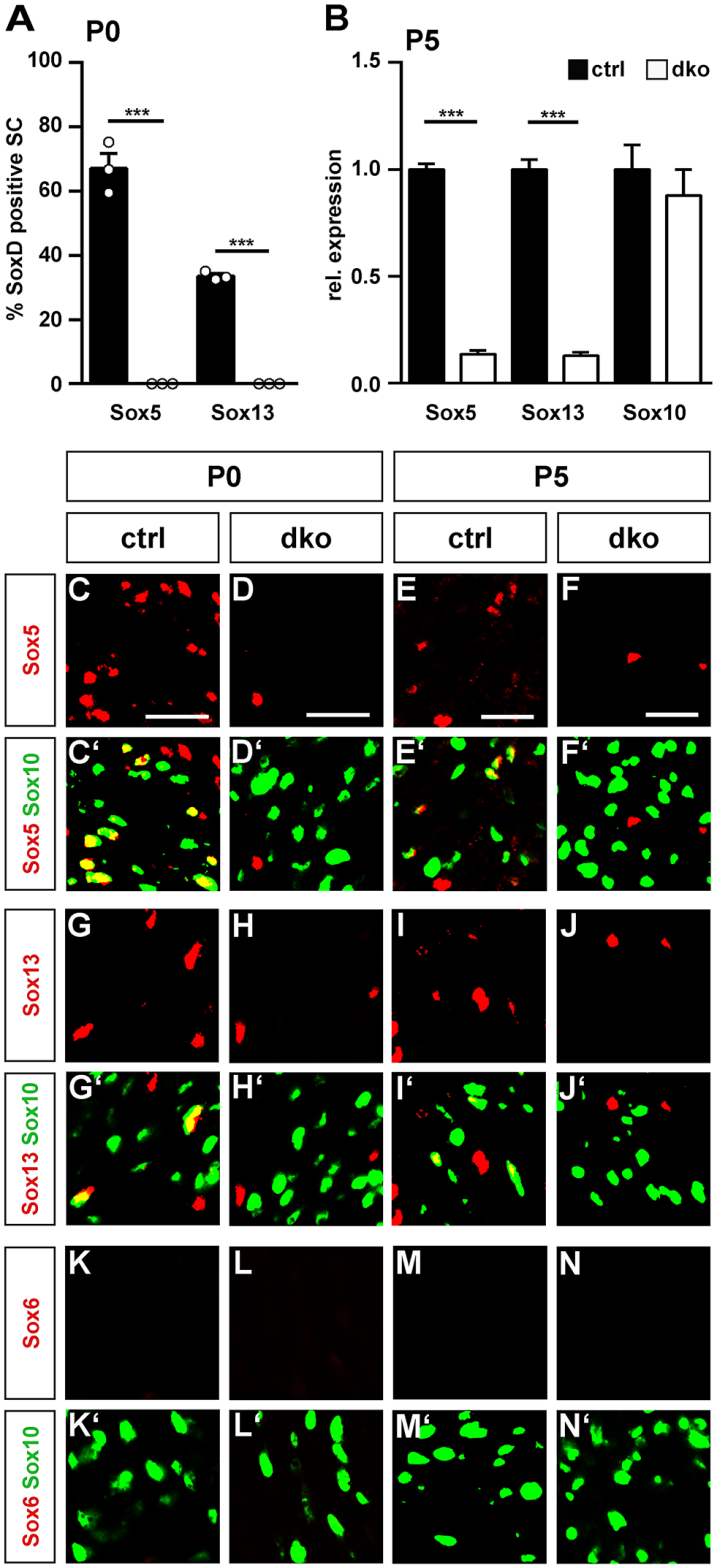
Analysis of SoxD deletion in Schwann cells of dko mice. (**A**) Quantification of the percentage of SoxD-positive Schwann cells at P0 in sciatic nerves of control (black bars) and dko (white bars) mice (n = 3; mean values ± SEM). (**B**) Quantification of the amount of *Sox5*, *Sox13* and *Sox10* transcripts in sciatic nerves of control and dko mice at P5 (n = 6) by qRT-PCR. Transcript levels in controls were set to 1 for each gene, those for dko mice expressed relative to it (± SEM). Statistical significance was determined by unpaired, two-tailed Student’s t-test (***P ≤ 0.001). For p-, t- and df-values, see Supplementary Fig. S3. (**C–N**) Co-immunohistochemistry was used to determine expression of Sox5 (**C–F**), Sox13 (**G–J**) and Sox6 (**K–N**) (all in red) in Sox10-positive Schwann cells (**C**′–**N**′, in green) of sciatic nerves of control (**C,C′,E,E′,G,G′,I,I′,K,K′,M,M′**) and dko (**D,D′,F,F′,H,H′,J,J′,L,L′,N,N′**) mice at P0 (**C,C′,D,D′,G,G′,H,H′,K,K′,L,L′**) and P5 (**E,E′,F,F′,I,I′,J,J′,M,M′,N,N′**). Orientation of nerves and magnified area were as shown in Fig. 1C,D. Scale bar, 25 µm.

Sox5 was present in nearly all Schwann cell nuclei at E11.5 and E14.5 (Fig. 1G,G′,H,H′). The number of Sox5-expressing Schwann cells amounted to 67 ± 5% by P0 (Figs. 1I,I', 2A) and to less than 30% by P5 (Fig. 1J,J′). At P28 and P60, Sox5 was no longer detectable (Fig. 1K,K′,L,L′). This argues that Sox5 is transiently expressed in developing Schwann cells and becomes downregulated upon terminal differentiation.

In a very similar pattern, Sox13 was detected in the vast majority of Sox10-positive Schwann cells at E11.5 and E14.5 (Fig. 1S,S′,T,T′). However, by P0 the fraction of Sox13-positive Schwann cells was already reduced to 34 ± 1% (Figs. 1U,U′, 2A). By P5, Sox13 had mostly disappeared from the Schwann cell lineage (Fig. 1V–X,V′–X′).

In contrast to this transient expression of Sox5 and Sox13 in developing Schwann cells, Sox6 was undetectable at all times (Fig. 1M–R,M′–R′). We conclude from these data, that SoxD expression in the Schwann cell lineage is restricted to Sox5 and Sox13 for a transient period before terminal differentiation.

### Sox5 and Sox13 were efficiently deleted from developing Schwann cells by *Dhh::Cre*

Considering that SoxD proteins frequently function in a redundant manner^4,5,7^, we decided to generate mouse mutants with joint Schwann cell-specific deletion of *Sox5* and *Sox13*. We chose to combine the floxed alleles of *Sox5* and *Sox13* with a *Dhh::Cre* transgene, as this transgene induces gene deletion in Schwann cell precursors with high efficiency and exhibits little expression outside the Schwann cell lineage^11^. The resulting mutant mice are referred to as dko mice.

Analysis of deletion rates in the sciatic nerve of these dko mice at birth indeed revealed that the number of Sox5 or Sox13 expressing Schwann cells was reduced to less than 5% (Fig. 2A). Levels of *Sox5* transcripts were also reduced to 16 ± 1% and those for Sox13 to 14 ± 2% in sciatic nerves of dko mice at P5 as compared to controls and determined by quantitative RT-PCR (qRT-PCR), whereas amounts of *Sox10* transcripts were not significantly different between genotypes (Fig. 2B). Immunohistochemistry on sciatic nerve sections at P0 and P5 confirmed that the remaining Sox5- and Sox13-expressing cells in the sciatic nerve of dko mice were not Schwann cells, as the signal did not overlap with staining for the pan-Schwann cell marker Sox10 (Fig. 2C–J,C′–J′). Importantly, Sox6 remained undetectable in sciatic nerve tissue of dko mice, arguing that there is no compensatory upregulation of Sox6 in the absence of Sox5 and Sox13 (Fig. 2K–N,K′–N′).

### Mice with a Schwann cell-specific deletion of Sox5 and Sox13 appeared phenotypically normal and exhibited normal expression of Schwann cell markers

**Figure 3:**
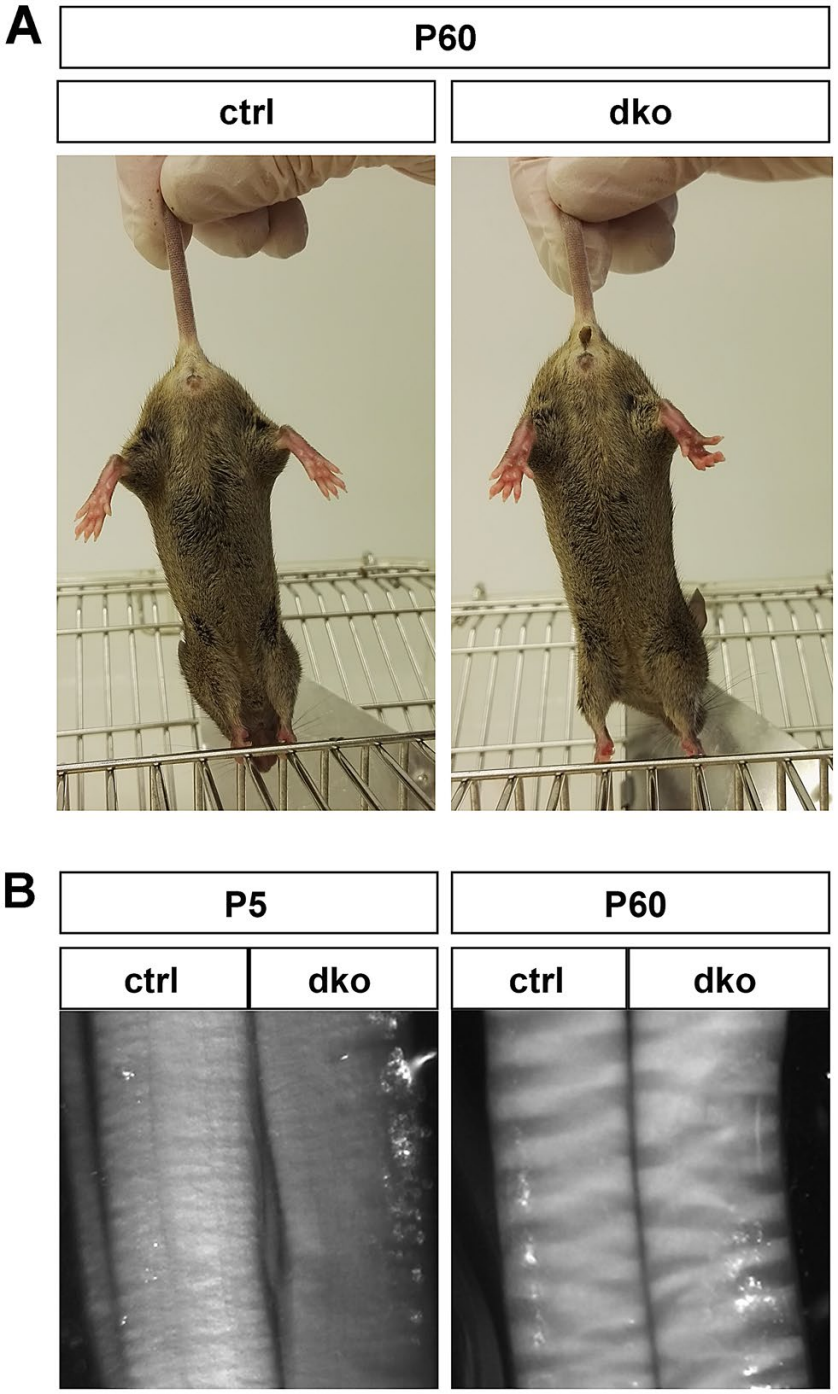
Behavioural phenotype and sciatic nerve appearance of dko mice. (**A**) Normal behaviour of dko mice at P60 compared to control mice in tail suspension test. (**B**) Appearance of sciatic nerve from control and dko mice at P5 and P60 after dissection.

Dko mice were obtained at expected Mendelian frequencies and did not exhibit any obvious phenotypic abnormalities during early postnatal times. At 2 months of age, motor coordination of dko mice was completely normal. There was no indication of gait disturbances, ataxia, or muscle weakness. When lifted at their tail, dko mice did not show typical signs of peripheral neuropathies such as hindlimb clasping (Fig. 3A). However, upon dissection at P5, the sciatic nerve was more translucent in dko mice than control mice (Fig. 3B). At later time points such as P60, this difference had disappeared.

**Figure 4:**
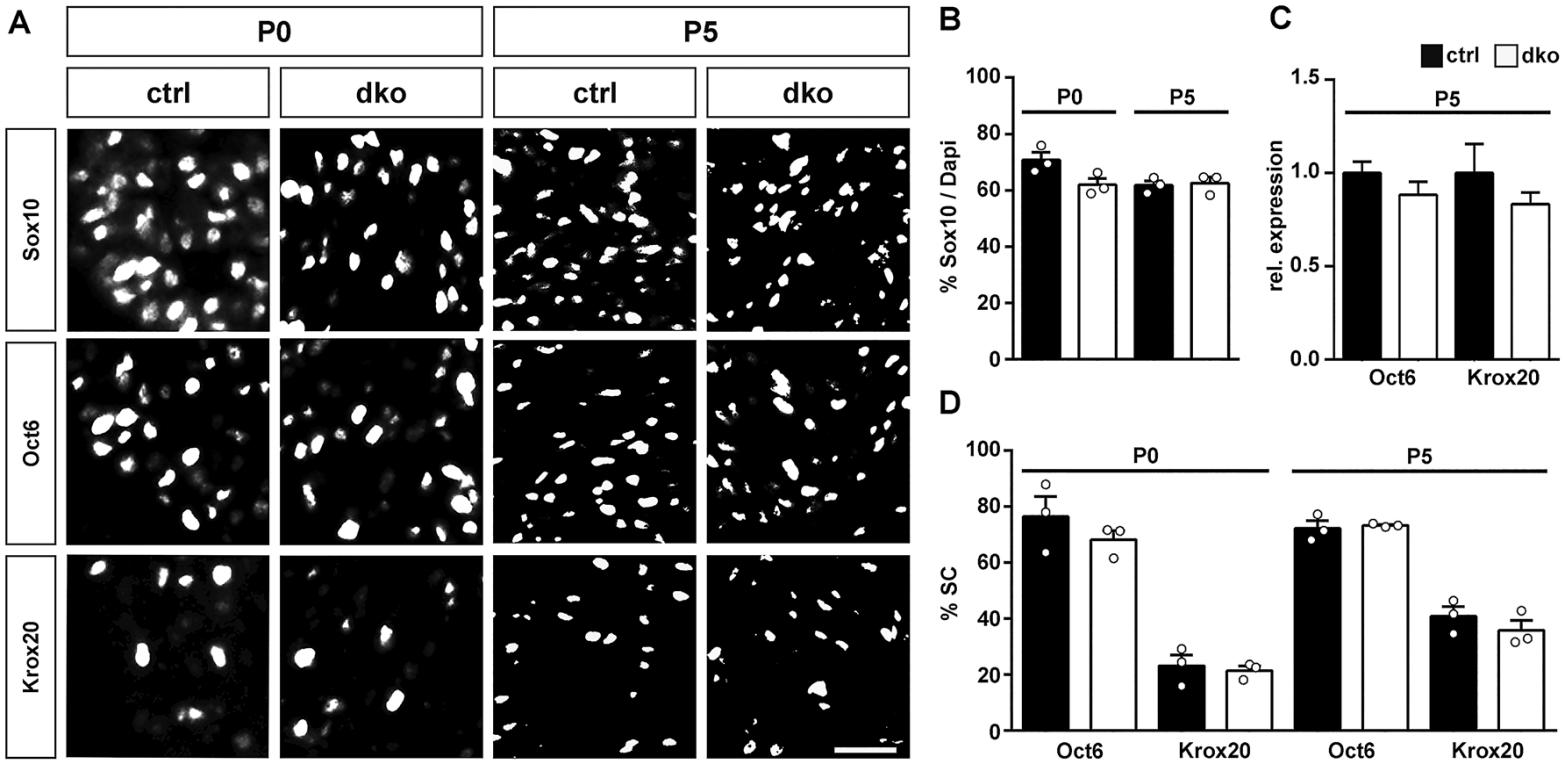
Expression of stage-specific transcription factors in sciatic nerves of dko mice at early postnatal stages. (**A**) Representative stainings on sciatic nerve sections of control and dko mice at P0 and P5 with antibodies directed against Sox10, Oct6 and Krox20. Scale bar, 25 µm. (**B**) Quantification of the percentage of Schwann cells among all cells in the sciatic nerve of control (black bars) and dko (white bars) mice at P0 and P5 (n = 3). (**C**) Quantification of the amount of *Oct6* and *Krox20* transcripts by qRT-PCR in sciatic nerves of control (black bars) and dko (white bars) mice at P5 (n = 6). Transcript levels in controls were set to 1 for each gene, those for dko mice expressed relative to it (± SEM). (**D**) Quantification of the percentage of Schwann cells that express Oct6 and Krox20 in the sciatic nerve of control and dko mice at P0 and P5 (n = 3). Numbers were not significantly different as determined by unpaired, two-tailed Student’s t-test. For p-, t- and df-values, see Supplementary Fig. S3. Bars represent mean values ± SEM.

As the different macroscopic appearance of the early postnatal nerve may be a consequence of altered Schwann cell development, we studied number and developmental status of Schwann cells in sciatic nerve tissue of dko mice. With Sox10 as a marker of all Schwann cells, we first determined whether Schwann cell numbers were changed in the absence of Sox5 and Sox13. However, this was not the case. Schwann cells represented 62 ± 2% of all cells in the mutant sciatic nerve at P0 and P5 as compared to 71 ± 3% at P0 and 62 ± 2% at P5 in the control (Fig. 4A,B). During early postnatal times, most Schwann cells furthermore expressed Oct6 as a marker of the promyelinating and early myelinating stage in control nerves (Fig. 4A). Again, we were unable to detect significant changes in dko mice. By qRT-PCR, *Oct6* transcript levels were very similar in sciatic nerves of control and dko mice (Fig. 4C). Furthermore, around two thirds of all Schwann cells labelled positive for Oct6 protein in both genotypes (Fig. 4A,D). Compared to Oct6, fewer Schwann cells expressed Krox20 as a marker of the myelinating stage in control nerves at P0 and levels should rise substantially at P5 (Fig. 4A). In control mice, the number of Krox20-positive Schwann cells indeed rose from 23 ± 4% at P0 to 41 ± 3% at P5 (Fig. 4A,D). In the mutant, Krox20-positive Schwann cells increased from 21 ± 2% of all Schwann cells at P0 to 36 ± 4% at P5 (Fig. 4A,D). The similarity in absolute numbers of Krox20-positive cells and in their share of the Schwann cell population was also reflected by the transcript levels in both genotypes. Amounts of *Krox20* transcripts were comparable in sciatic nerves of control and dko mice at P5 (Fig. 4C). These studies therefore led us to conclude that Schwann cell number and lineage progression are not substantially altered in the absence of Sox5 and Sox13 during early postnatal stages.

**Figure 5:**
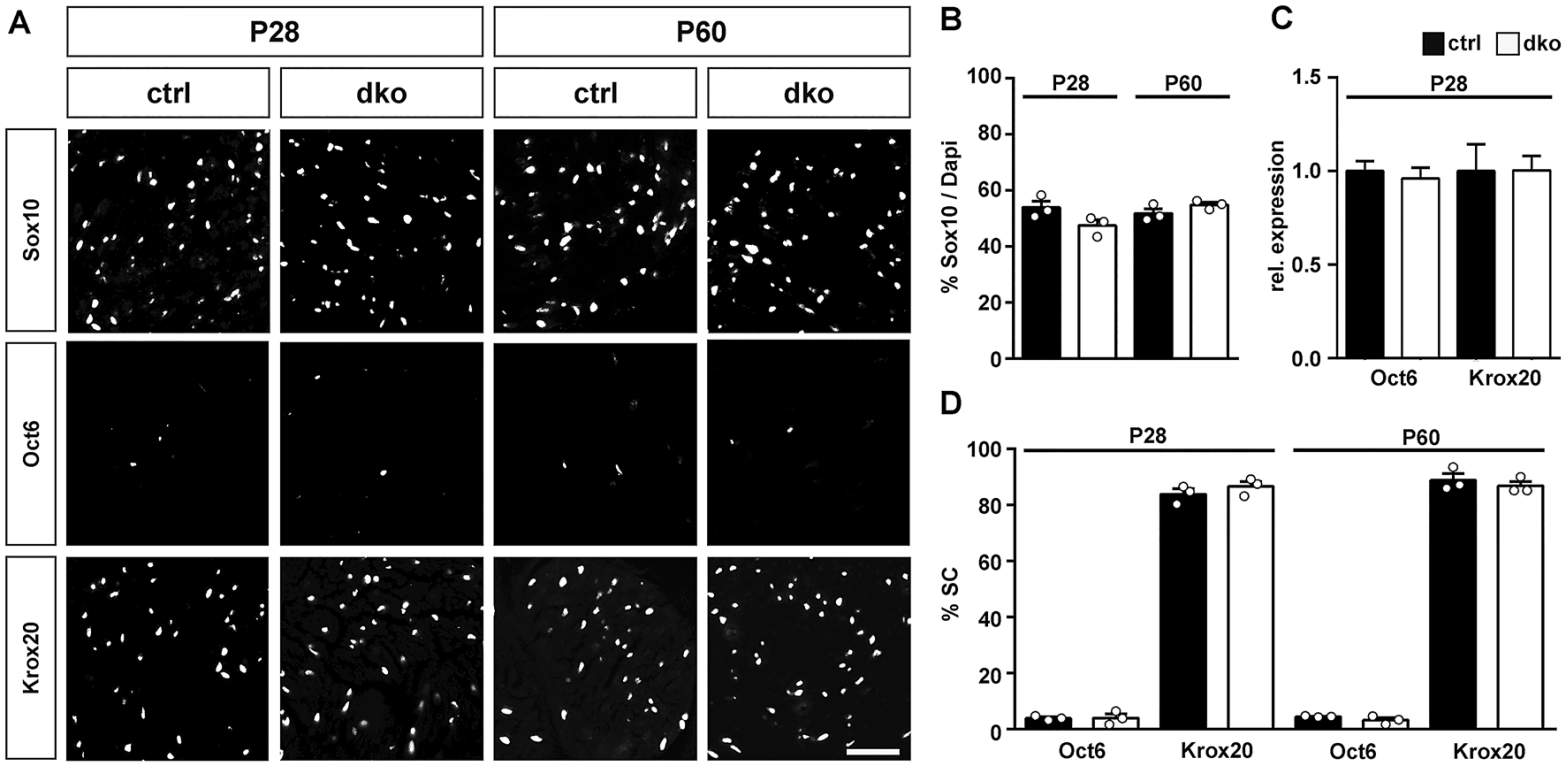
Expression of stage-specific transcription factors in sciatic nerves of adolescent and adult dko mice. (**A**) Representative stainings on sciatic nerve sections of control and dko mice at P28 and P60 with antibodies directed against Sox10, Oct6 and Krox20. Scale bar, 25 µm. (**B**) Quantification of the percentage of Schwann cells among all cells in the sciatic nerve of control (black bars) and dko (white bars) mice at P28 and P60 (n = 3). (**C**) Quantification of the amount of *Oct6* and *Krox20* transcripts by qRT-PCR in sciatic nerves of control (black bars) and dko (white bars) mice at P28 (n = 4). Transcript levels in controls were set to 1 for each gene, those for dko mice expressed relative to it (± SEM). (**D**) Quantification of the percentage of Schwann cells that express Oct6 and Krox20 in the sciatic nerve of control and dko mice at P28 and P60 (n = 3; mean values ± SEM). Numbers were not significantly different as determined by unpaired, two-tailed Student’s t-test. For p-, t- and df-values, see Supplementary Fig. S3.

When marker gene studies were extended to P28 and P60, overall numbers of Sox10-positive Schwann cells remained comparable between genotypes (Fig. 5A,B). As expected, the number of Oct6-positive Schwann cells (and therefore their percentage among all Schwann cells) dropped substantially in older mice, whereas the number of Krox20-positive cells increased because the majority of Schwann cells entered the myelinating stage characterized by Krox20 expression (Fig. 5A,C,D). Again, the number of Oct6- and Krox20-positive Schwann cells and their relative contribution to the overall Schwann cell population were very similar in control and dko mice (Fig. 5A,D). This was also reflected in comparable levels of *Oct6* and *Krox20* transcripts in sciatic nerves of both genotypes at P28 (Fig. 5C).

### Mice with a Schwann cell-specific deletion of Sox5 and Sox13 exhibited a mild and transient delay in peripheral myelination

**Figure 6:**
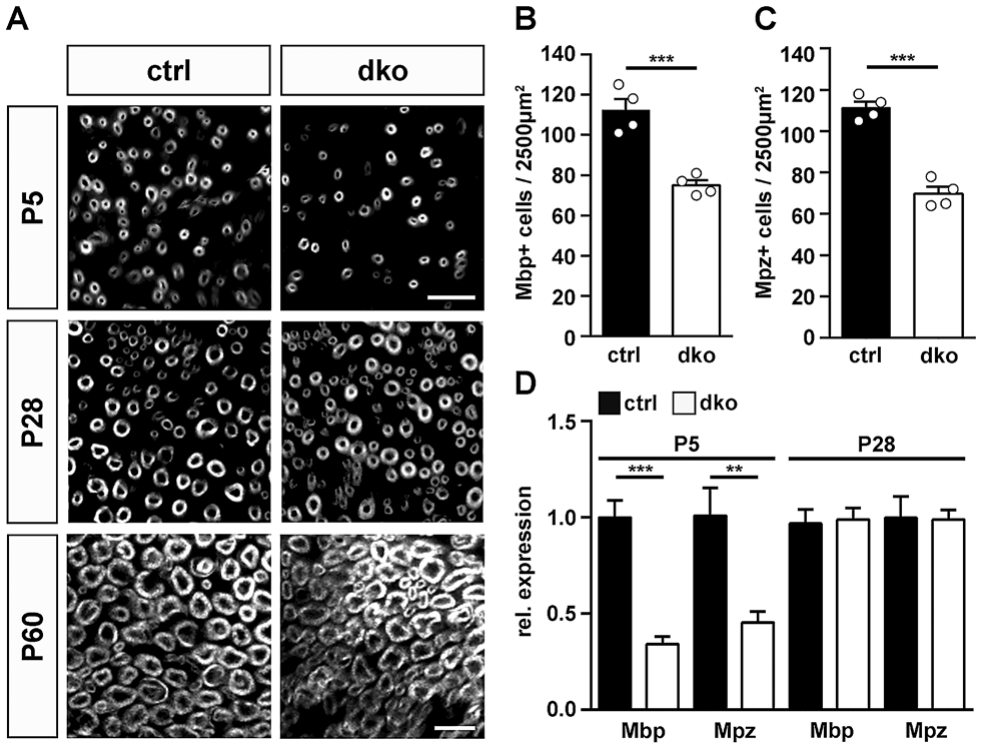
Myelin gene expression in sciatic nerves of dko mice. (**A**) Immunohistochemical detection of Mbp in sections of sciatic nerves of control and dko mice at P5, P28 and P60. Scale bar, 10 µm. (**B,C**) Quantification of Mbp- (**B**) and Mpz-positive (**C**) Schwann cells in the sciatic nerve of control (black bars) and dko (white bars) mice at P5 (n = 4; mean values ± SEM). (**D**) Quantification of the amount of *Mbp* and *Mpz* transcripts by qRT-PCR in sciatic nerves of control (black bars) and dko (white bars) mice at P5 and P28 (n = 6). Transcript levels in controls were set to 1 for each gene, those for dko mice expressed relative to it (± SEM). Statistical significance was determined by unpaired two-tailed Student’s t-test (**P ≤ 0.01; ***P ≤ 0.001). For p-, t- and df-values, see Supplementary Fig. S3.

**Figure 7:**
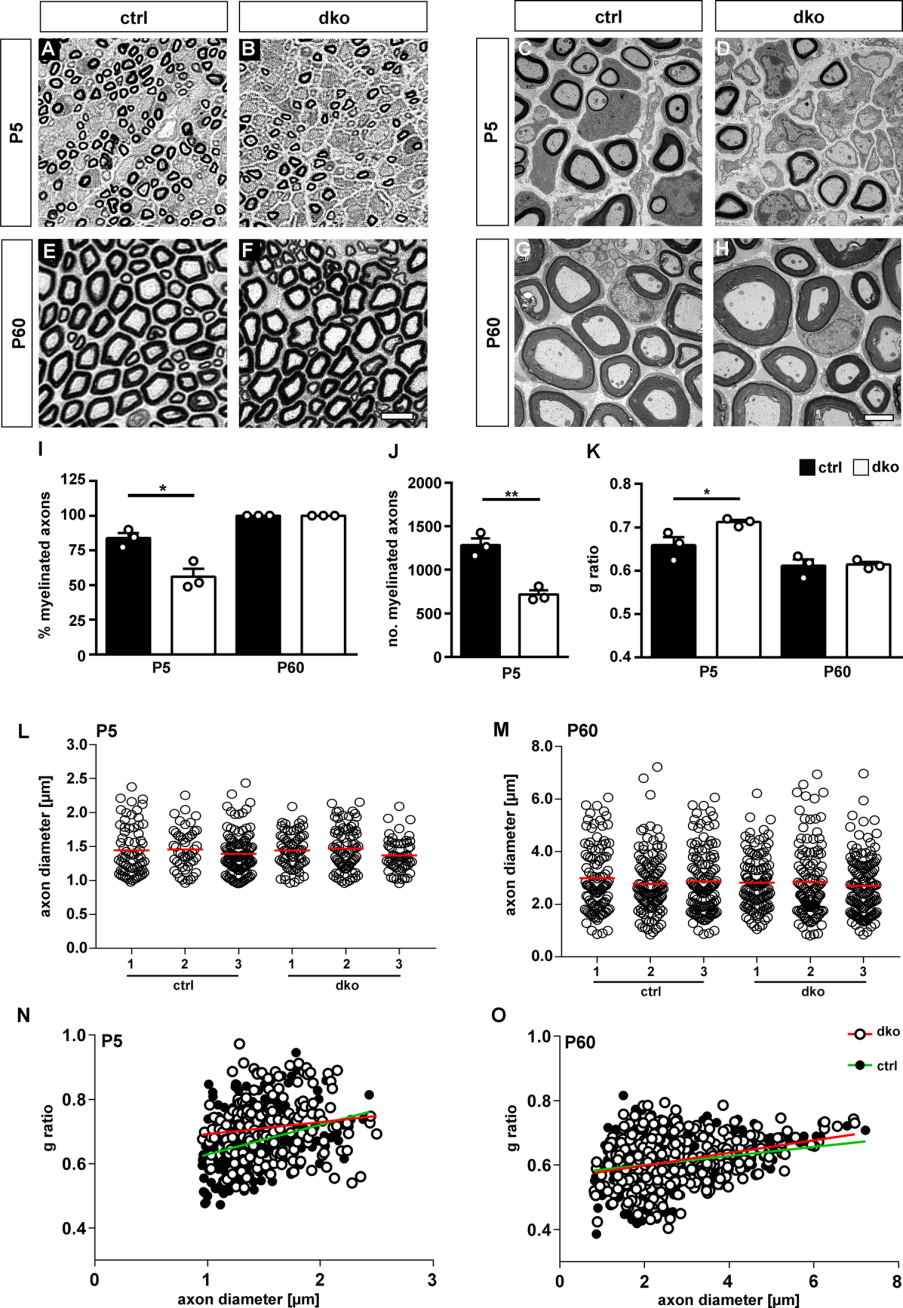
Histology and ultrastructure of sciatic nerves of dko mice. (**A–H**) Representative PPD stainings (**A,B,E,F**) and electron microscopic pictures (**C,D,G,H**) of sections of sciatic nerve tissue of control and dko mice at P5 and P60. Scale bars, 10 µm (left) and 2 µm (right). (**I**) Percentage of myelinated axons in sections of sciatic nerves from control and dko mice at P5 and P60 (n = 3; ± SEM). (**J**) Absolute number of myelinated axons per section in sciatic nerves from control (black bars) and dko (white bars) mice at P5 (n = 3; ± SEM). (**K**) Determination of average g ratios of myelinated axons in sections of sciatic nerves from control and dko mice at P5 and P60 (n = 3; ± SEM). Statistical significance was determined by unpaired, two-tailed Student’s t-test (*P ≤ 0.05; **P ≤ 0.01). For p-, t- and df-values, see Supplementary Fig. S3. (**L,M**) Diameters of myelinated axons in sciatic nerves from three individual control and dko mice at P5 (**L**) and P60 (**M**). Red lines depict mean values. (**N,O**) Scatter blot showing single g ratios of 100–150 myelinated axons in sciatic nerves from control (black dots) and dko (white dots) mice at P5 (**N**) and P60 (**O**). Green and red lines show linear regression.

To study myelination in closer detail, we performed immunohistochemical stainings for the myelin proteins Mbp and Mpz (for representative Mbp picture see Fig. 6A). At P5, fewer Mbp- and Mpz-positive cells were detected in sciatic nerves of dko mice as compared to controls and quantification revealed that the reduction in dko mice amounted to 33–37% (Fig. 6B,C). At P28 and P60, this difference was no longer detectable (Fig. 6A). In support of the transient reduction in myelinating Schwann cells during early postnatal times, amounts of *Mbp* and *Mpz* transcripts were reduced in dko sciatic nerves relative to controls at P5, while this reduction was no longer visible at P28 (Fig. 6D). PPD stainings on semithin sections of sciatic nerve tissue at P5 and P60 confirmed the immunohistochemical data. Myelinated axons were present in both genotypes at the two time points of analysis (Fig. 7A,B,E,F). Myelin sheaths in sciatic nerves of dko mice furthermore exhibited a normal ultrastructure as determined by electron microscopy (Fig. 7C,D,G,H). Unusual structures such as myelin outfoldings or myelin debris were not observed in substantial amounts in sciatic nerves of dko mice. However, both PPD stainings and electron micrographs revealed a higher number of unmyelinated large calibre axons in sciatic nerve tissue of dko mice at P5 (compare Fig. 7A,C to B,D). Quantification demonstrated that 84 ± 4% of the large calibre axons were already surrounded by myelin at P5 in control mice in contrast to 56 ± 6% in dko mice (Fig. 7I). As a consequence, the absolute number of myelinated axons per section was substantially higher in sciatic nerves of control mice at P5 than in age-matched dko littermates (Fig. 7J). This was not due to overall changes in the diameter of peripheral axons or their size distribution (Fig. 7L, Supplementary Fig. S1A). A closer look at the unmyelinated axons revealed that they were in a 1:1 relationship with Schwann cells and thus had progressed into the promyelinating stage (Fig. 7B,D). Those axons that were myelinated at P5 exhibited an average g ratio of 0.71 ± 0.01 in dko mice as compared to a g ratio of 0.66 ± 0.2% in control mice (Fig. 7K). The higher g ratio is indicative of a thinner myelin sheath. Among all myelinated axons, those with a smaller diameter appeared to be more strongly affected in the dko mutant (Fig. 7N).

Differences in overall appearance and the number of unmyelinated large calibre axons had disappeared at P60 (Fig. 7E–I,M, Supplementary Fig. S1B). The g ratio was determined as 0.61 ± 0.01 in both genotypes (Fig. 7K,O, Supplementary Fig. S1B). This argues that myelination deficits in sciatic nerves of dko mice were transient. The organization of small calibre axons with Schwann cells in Remak bundles appeared normal at all analysed time points. We therefore conclude that peripheral myelination by Schwann cells exhibits a mild and transient delay in the combined absence of Sox5 and Sox13.

## Discussion

In this study we have analysed the expression and function of SoxD proteins in Schwann cell development and peripheral myelination. Previous studies had shown that early neural crest cells and several neural crest derivatives such as cells of the melanocyte lineage express Sox5^9,10^. Therefore, prominent expression of Sox5 in the neural crest-derived Schwann cells was not unexpected. Additionally, we detected Sox13 in developing Schwann cells. Both SoxD proteins were furthermore found to be transiently expressed and downregulated shortly after the onset of terminal differentiation. Despite their downregulation in differentiating Schwann cells, combined deletion of Sox5 and Sox13 led to a mild and transient delay in myelination. The extent of the delay is roughly in the same range as the one reported for mice with Schwann cell-specific overexpression of Sox4 or Schwann cell-specific loss of Ebf2, but substantially less than observed in mice with deficiencies in POU domain transcription factors Oct6 and Brn2 or in Nfat signaling^11–14^. First results furthermore suggest that SoxD gene-deficient Schwann cells in the early postnatal sciatic nerve exhibit reduced expression levels of Hes1 and Sox2, but not c-Jun (Supplementary Fig. S2). These factors represent immaturity factors that counteract Schwann cell differentiation^15–17^. It is tempting to speculate that the observed reduction in immaturity factors may help Schwann cells to overcome the transient myelination defect.

The role of SoxD proteins during Schwann cell development contrasts strongly with previous findings for oligodendroglial cells. In this glial cell type of the CNS, all three SoxD proteins were found to be expressed^5–7^. Sox6 is the SoxD protein with the strongest expression and most important contribution. In this respect, it deserves to be noted that Sox6 is not at all expressed in Schwann cells. More importantly, the overall effects of SoxD proteins on Schwann cell development are much milder than on oligodendrocyte development and their impact on differentiation is reversed in the two cell types with a differentiation-promoting role of SoxD proteins in Schwann cells and a differentiation-repressing role in oligodendrocytes. Such differences are surprising but correspond to other circumstances where transcription factors with relevance for development in one type of myelinating glia are not expressed in the other or have different functions in the two glial cell types^18^.

In oligodendroglial cells and melanocytes but also in other cell types such as chondrocytes, SoxD proteins have been shown to work by functionally interacting with SoxE proteins such as Sox9 or Sox10^4,5,19^. This functional interaction may be antagonistic and therefore inhibiting SoxE function as seen in oligodendrocytes and melanocytes^5,10^ or auxiliary and thus amplifying SoxE function as described for chondrocytes^19–21^. However, SoxD proteins have also been shown to have SoxE-independent functions, for instance during muscle development or oncogene-driven transformation of brain stem cells^22,23^. Currently, we do not know whether SoxD function during Schwann cell development depends on Sox10 as the only expressed SoxE protein.

Our analysis shows that Schwann cells progress normally into the promyelinating stage without SoxD proteins, but then require extra time to initiate myelination. Still, we do not see any alterations in the temporal appearance or amounts of the transcription factors that are known to regulate the later stages of Schwann cell development such as Oct6 and Krox20. Therefore, it is unlikely that SoxD proteins exert their effect on myelin gene expression by influencing Krox20 levels. Because of the expression pattern of SoxD proteins, we favour a model in which SoxD proteins exert their effect on Schwann cell development in an indirect and complex manner. The detected changes in Hes1 and Sox2 expression attest to that. We assume that SoxD proteins control features in early stages of Schwann cell development that facilitate differentiation later on in myelinating Schwann cells. What these features are, is currently unknown as are direct target genes for SoxD proteins.

During the early stages of Schwann cell development, several other transcription factors are expressed that function as important developmental regulators in other systems but have only limited influence on Schwann cell development. Among others, these include c-Jun and Sox2^15,16^. Intriguingly, these factors become essential during remyelination following a demyelinating event or injury of the peripheral nerve^24,25^. Therefore, it will be interesting to study SoxD proteins during remyelination and look for potential functions.

## Methods

### Husbandry and breeding of mice

Mice carrying floxed alleles for *Sox5* and *Sox13* were bred with mice expressing a *Dhh:Cre* transgene^6,11,26^. Genotyping was performed as described. All mice were on a mixed C3H × C57Bl/6J background and kept under standard housing conditions with 12:12 h light–dark cycles and continuous access to food and water. Timed matings were set up to generate litters at embryonic day 11.5 and 14.5 (E11.5 and E14.5). Spinal nerves of embryos or sciatic nerves of pups and mice of both sexes were used to prepare RNA or were processed for immunohistochemical studies as described^27^. Experiments were in accordance with animal welfare laws, complied with ARRIVE guidelines and were approved by the responsible local committees and government bodies (University, Veterinäramt Stadt Erlangen & Regierung von Unterfranken, TS-00/12 Biochemie II).

### Immunohistochemical analysis

For immunohistochemistry, embryos and dissected sciatic nerve tissue underwent fixation in 4% paraformaldehyde, cryoprotection in 30% sucrose, embedding in OCT compound, and sectioning on a Leica CM3050S cryotome. Immunohistochemistry was performed on 10-µm-thick sections using the following primary antibodies: anti-Sox10 goat antiserum (1:1000 dilution, RRID:AB_2891326)^28^, anti-Krox20 guinea pig antiserum (1:1000 dilution, RRID:AB_2891327)^29^, anti-Sox6 guinea pig antiserum (1:1000 dilution, RRID:AB_2891329)^5^, anti-Sox5 guinea pig antiserum (1:500 dilution, RRID:AB_2891328)^5^, anti-Sox5 rabbit antiserum (1:4000 dilution, gift of J. Barbas, Instituto Cajal, Madrid, Spain)^9^, anti-Sox13 rabbit antiserum (1:1000 dilution, RRID:AB_2891330)^6^, anti-Oct6 rabbit antiserum (1:2000 dilution, RRID:AB_2891333)^30^, anti-neurofilament NF-H chicken antibodies (EnCor Biotechnology Cat# CPCA-NF-H, Lot: 3-1003, 1:2000 dilution RRID:AB_2149761) and anti-MBP rat monoclonal antibody (Bio-Rad, #MCA409S, Lot #210610, 1:500 dilution, RRID:AB_325004). Cy3- or Alexa488-coupled secondary antibodies (Thermo Fisher Cat# A21202 and A21206, Jackson ImmunoResearch Labs Cat# 706-545-148, 715-165-150, 711-165-152 and 706-165-148) were used for fluorescence labeling. Nuclei were counterstained with 4′,6-diamidino-2-phenylindole (DAPI, Sigma Cat# D9542). Fluorescent signals were documented with a Leica DMI 6000B inverted microscope (Leica) equipped with a DFC 360FX camera (Leica). Marker quantifications focused on the tibial branch of the sciatic nerve at upper thigh levels. For each sciatic nerve, four sections were counted and used to determine the mean value for each replicate. Whole nerve sections were quantified for nuclear markers and four arbitrarily placed 50 × 50 µm squares per section for Mbp and Mpz stainings. Error bars represent SEM.

### Histology and electron microscopy

After dissection, sciatic nerves of control and genetically altered mice were incubated in cacodylate-buffered fixative containing 2.5% paraformaldehyde and 2.5% glutaraldehyde before incubation in cacodylate-buffered 1% osmium ferrocyanide, dehydration, embedding in Epon resin, sectioning and staining with uranyl acetate and lead citrate. Para-phenylene-diamine (PPD) stainings were performed on 1 µm semithin sections. In brief, 1% PPD (Carl Roth, Karlsruhe, Germany) in 98% ethanol was freshly prepared and stored at daylight for 3 days until the solution had darkened. Sections were stained for 30 min at room temperature and differentiated with changes of 100% ethanol^31^. Stainings were analysed by light microscopy and documented using a Leica DMR microscope. From these pictures the number of myelinated axons per section with a calibre larger than 1 µm was determined. For electron microscopy, 50 nm ultrathin sections were generated and examined on a Zeiss Libra electron microscope (Carl Zeiss, Inc.). From these sections the corresponding g ratio (as the ratio of the inner axonal diameter and the outer, myelinated, axonal diameter) was determined. For g ratio determination, measurements were along the largest diameter of each axon. Between 100 and 150 axons were counted using at least 10 representative sections per animal. Error bars represent SEM.

### RNA preparation, reverse transcription and quantitative PCR

Sciatic nerves were dissected and dissocated using gentleMACS™ Dissociator (Miltenyi Biotec) before addition of Trizol reagent and RNA preparation according to the manufacturer’s protocol (Invitrogen Cat# 15596018). RNA samples were reverse transcribed and used to determine expression levels by quantitative PCR on a Bio-Rad CFX96 Real-Time PCR Detection System using the PowerUp™ SYBR™ Green Master Mix (Thermo Fisher Scientific, Darmstadt, Germany). The following primer pairs were used: 5′-GTTCGTGTACTGCGGCAAGA-3′ and 5′-ACAGGATTCATGGCACACACC-3′ for *Rpl8*, 5′-TGGCAATGGGATCAGGGAAC-3′ and 5′-CCATCTGCTGACGCTGTTTC-3′ for *Sox5*, 5′AGAAGCTGCTGTCCAGTGAC-3′ and 5′-CTTCTCAAACAGCATGGCGG-3′ for *Sox13*, 5′-CTTCTCAAACAGCATGGCGG-3′ and 5′-CATGTGAAAGTGCCGTTGTC-3′ for *Mpz*, 5′-CCAAGTTCACCCCTACTCCA-3′ and 5′-TAAGTCCCCGTTTCCTGTTG-3′ for *Mbp*, 5′-GTTCTCGCAGACCACCATCT-3′ and 5′-GGCTTGGGACACTTGAGAAA-3′ for *Oct6*, 5′-TGGCAATGGGATCAGGGAAC-3′ and 5′-AAGATGCCCGCACTCACAAT-3′ for *Krox20,* 5′-ACAGCAGCAGGAAGGCTTCT-3′ and 5′-TGTCCTCAGTGCGTCCTTAG-3′ for *Sox10.* Levels of all transcripts were normalized to *Rpl8* levels. Normalized levels for a particular transcript in control samples were arbitrarily set to 1 and values for samples from dko mice were set in relation to it. Error bars represent SEM.

### Statistical analysis

Whenever possible, experiments and analyses were carried out in a blinded fashion. No sample calculation was performed. Sample size was set to n = 3–6 as common for such studies, if not otherwise stated. Sample size did not differ between beginning and end of experiments. Results from independent specimens were treated as biological replicates. To determine whether differences in cell numbers or amounts were statistically significant, a Student’s t test was performed (*P ≤ 0.05; **P ≤ 0.01, ***P ≤ 0.001) using Graph Pad Prism 6 (GraphPad software, La Jolla, CA, USA).

## Supplementary Information

Supplementary Information.

## Data Availability

All data generated and analysed during this study are included in this article or its Supplemental Material.
