# Supplementary material for: SoxD transcription factor deficiency in Schwann cells delays myelination in the developing peripheral nervous system
Source: Sci Rep. 2021 Jul 7;11:14044. doi: 10.1038/s41598-021-93437-9 (PMC8263579; doi:10.1038/s41598-021-93437-9)
Supplement: Supplementary file 1 — Supplementary Information. [file 41598_2021_93437_MOESM1_ESM.pdf]

# Supplementary Information

**for**

**SoxD transcription factor deficiency in Schwann cells delays myelination in the developing peripheral nervous system**

By

Ella Ittner, Anna C. Hartwig, Olga Elsesser, Hannah M. Wüst, Franziska Fröb, Miriam Wedel, Margit Schimmel, Ernst R. Tamm, Michael Wegner, Elisabeth Sock

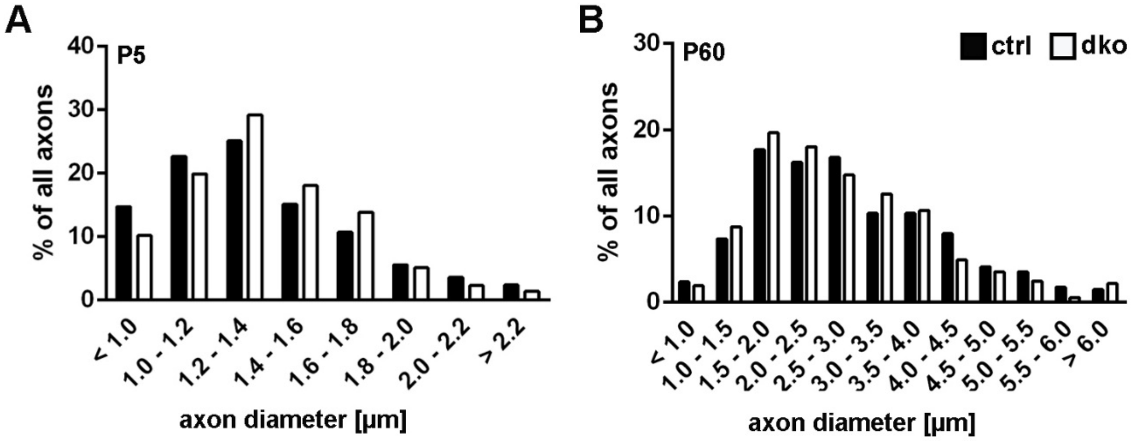


**Supplementary Figure 1:** Relative distribution of diameters of myelinated axons in sciatic nerves from control and dko mice at P5 (A) and P60 (B). The dataset corresponds to the one used for Fig. 7L,M. In the presented histograms, axons from individual control and dko mice (n = 3) were pooled and binned as indicated on the y-axis.

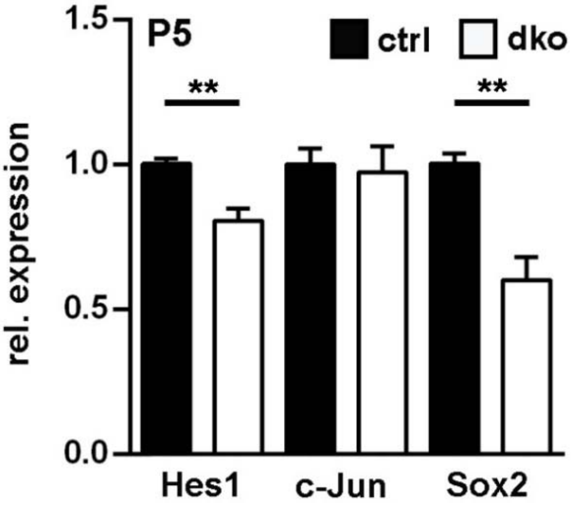


**Supplementary Figure 2:** Quantification of the amount of *Hes1*, *c-Jun* and *Sox2* transcripts in sciatic nerves of control and dko mice at P5 by quantitative RT-PCR. Transcript levels in controls were set to 1 for each gene and those for dko mice expressed relative to it (n = 4; mean values ± SEM). Statistical significance was determined by unpaired, two-tailed Student's t-test (***, P ≤0.001). For p-,t- and df-values, see Supplementary Fig. 3.

The following primers were used:

*Hes1:* 5'-GTGAAGCACCTCCGGAACCT-3' and 5'-GGTAGGTCATGGCGTTGATC-3'

*c-Jun*: 5'-ACCCCCACTCAGTTCTTGTG-3' and 5'-AGTTGCTGAGGTTGGCGTAG-3'

*Sox2*: 5'-CACAACTCGGAGATCAGCAA-3' and 5'-CTCCGGGAAGCGTGTACTTA-3'

| Figure | Age/Factor | Statistical test | Group | p-value | t-value | df |
|---|---|---|---|---|---|---|
| Fig2A | P0 Sox5 | t-test (two-tailed) | ctrl vs. dko | 0.0001 | 14.75 | 4 |
| | P0 Sox13 | t-test (two-tailed) | ctrl vs. dko | < 0.0001 | 44.21 | 4 |
| Fig2B | P5 Sox5 | t-test (two-tailed) | ctrl vs. dko | < 0.0001 | 26.97 | 10 |
| | P5 Sox13 | t-test (two-tailed) | ctrl vs. dko | < 0.0001 | 18.06 | 10 |
| | P5 Sox10 | t-test (two-tailed) | ctrl vs. dko | 0.4820 | 0.730 | 10 |
| Fig4B | P0 Sox10 | t-test (two-tailed) | ctrl vs. dko | 0.0660 | 2.508 | 4 |
| | P5 Sox10 | t-test (two-tailed) | ctrl vs. dko | 0.8044 | 0.265 | 4 |
| Fig4C | P5 Oct6 | t-test (two-tailed) | ctrl vs. dko | 0.2398 | 1.250 | 10 |
| | P5 Krox20 | t-test (two-tailed) | ctrl vs. dko | 0.3462 | 0.9884 | 10 |
| Fig4D | P0 Oct6 | t-test (two-tailed) | ctrl vs. dko | 0.3466 | 1.066 | 4 |
| | P0 Krox20 | t-test (two-tailed) | ctrl vs. dko | 0.6998 | 0.414 | 4 |
| | P5 Oct6 | t-test (two-tailed) | ctrl vs. dko | 0.7192 | 0.386 | 4 |
| | P5 Krox20 | t-test (two-tailed) | ctrl vs. dko | 0.3624 | 1.027 | 4 |
| Fig5B | P28 Sox10 | t-test (two-tailed) | ctrl vs. dko | 0.1017 | 2.117 | 4 |
| | P60 Sox10 | t-test (two-tailed) | ctrl vs. dko | 0.1846 | 1.601 | 4 |
| Fig5C | P28 Oct6 | t-test (two-tailed) | ctrl vs. dko | 0.6340 | 0.501 | 6 |
| | P28 Krox20 | t-test (two-tailed) | ctrl vs. dko | 0.9766 | 0.036 | 6 |
| Fig5D | P28 Oct6 | t-test (two-tailed) | ctrl vs. dko | 0.9563 | 0.058 | 4 |
| | P28 Krox20 | t-test (two-tailed) | ctrl vs. dko | 0.3519 | 1.053 | 4 |
| | P60 Oct6 | t-test (two-tailed) | ctrl vs. dko | 0.2155 | 1.470 | 4 |
| | P60 Krox20 | t-test (two-tailed) | ctrl vs. dko | 0.5069 | 0.728 | 4 |
| Fig6B | P5 Mbp | t-test (two-tailed) | ctrl vs. dko | 0.0009 | 6.091 | 6 |
| Fig6C | P5 Mpz | t-test (two-tailed) | ctrl vs. dko | < 0.0001 | 9.945 | 6 |
| Fig6D | P5 Mbp | t-test (two-tailed) | ctrl vs. dko | < 0.0001 | 6.730 | 10 |
| | P5 Mpz | t-test (two-tailed) | ctrl vs. dko | 0.0049 | 3.593 | 10 |
| | P28 Mbp | t-test (two-tailed) | ctrl vs. dko | 0.8386 | 0.2090 | 10 |
| | P28 Mpz | t-test (two-tailed) | ctrl vs. dko | 0.9463 | 0.0691 | 10 |
| Fig7I | P5 | t-test (two-tailed) | ctrl vs. dko | 0.0150 | 4.091 | 4 |
| Fig7J | P5 | t-test (two-tailed) | ctrl vs. dko | 0.0033 | 6.268 | 4 |
| Fig7K | P5 | t-test (two-tailed) | ctrl vs. dko | 0.0495 | 2.787 | 4 |
| | P60 | t-test (two-tailed) | ctrl vs. dko | 0.8905 | 0.147 | 4 |
| SuppFig2 | Hes1 | t-test (two-tailed) | ctrl vs. dko | 0.0056 | 4.213 | 6 |
| | c-Jun | t-test (two-tailed) | ctrl vs. dko | 0.8027 | 0.2611 | 6 |
| | Sox2 | t-test (two-tailed) | ctrl vs. dko | 0.0034 | 4.677 | 6 |

**Supplementary Figure 3:** Summary of the p-,t- and df-values for the unpaired t tests performed in the study.
